# Exploring the Potential Mechanism of Shennao Fuyuan Tang for Ischemic Stroke Based on Network Pharmacology and Molecular Docking

**DOI:** 10.1155/2021/6015702

**Published:** 2021-09-23

**Authors:** Jia Min Li, Zhen Ni Mu, Tian Tian Zhang, Xin Li, Yan Shang, Guo Heng Hu

**Affiliations:** ^1^Affiliated Hospital of Hunan University of Traditional Chinese Medicine, Changsha, Hunan, China; ^2^Graduate School of Hunan University of Traditional Chinese Medicine, Changsha, Hunan, China

## Abstract

**Methods:**

Screen the biologically active components and potential targets of SNFYT through Traditional Chinese Medicine Systems Pharmacology (TCMSP), Traditional Chinese Medicines Integrated Database (TCMID), and related literature. In addition, DrugBank, OMIM, DisGeNET, and the Therapeutic Target Database were searched to explore the therapeutic targets of IS. The cross-targets of SNFYT potential targets and IS treatment targets were taken as candidate gene targets, and GO and KEGG enrichment analyses were performed on the candidate targets. On this basis, the SNFYT-component-target network and protein-protein interaction (PPI) network were constructed using Cytoscape 3.7.2. Finally, AutoDock was used to verify the molecular docking of core components and core targets.

**Results:**

We screened out 95 potentially active components and 143 candidate targets. SNFYT-component-target network, PPI network, and Cytoscape analysis identified four core active ingredients and 14 core targets. GO enrichment analyzed 2333 biological processes, 79 cell components, and 149 molecular functions. There are 170 KEGG-related signal pathways (*P* < 0.05), including the IL-17 signal pathway, TNF signal pathway, and HIF-1 signal pathway. The molecular docking results of the core components and the core targets showed good binding power.

**Conclusions:**

SNFYT may achieve the effect of treating ischemic stroke through its anti-inflammatory effect through a signal pathway with core targets as the core.

## 1. Introduction

Ischemic stroke is a kind of disease caused by various causes of local brain tissue blood supply obstruction, which leads to brain tissue ischemia, hypoxia, and necrosis and then produces a variety of clinical neurological impairment symptoms. Stroke is one of the most common causes of death and disability worldwide [1]. According to epidemiological data, stroke affects one in every four people in their lifetime and is the second leading cause of death and the third leading cause of disability among adults worldwide [2]. It is particularly noteworthy that ischemic stroke accounts for the highest proportion among all stroke types, up to 70%–80% [3]. In recent decades, the treatment methods of ischemic stroke have constantly been developing, but unfortunately, the current clinical evidence level of drugs for the treatment of ischemic stroke is generally not high [4, 5]. Recombinant tissue plasminogen activator (rt-PA) is the only FDA-approved drug for acute ischemic stroke treatment. However, the limited time window [6, 7] and the tendency to induce hemorrhagic transformation [8] have severely limited the clinical use of thrombolysis, and only a small number of people can benefit from it, even under advanced health care conditions [9]. Therefore, given the complex pathophysiological links of ischemic stroke, it has become an urgent clinical problem to seek treatment that can improve the symptoms of clinical neurological impairment in patients.

In China, Traditional Chinese Medicine (TCM) was widely used more than 2,000 years ago to treat various diseases, including stroke. Different from modern medicine, the treatment of ischemic stroke by traditional Chinese medicine is not targeted at one or several action targets but multisystem and multilevel regulation of the human body. In addition, compared with thrombolytic drugs and anticoagulant drugs, the side effects of traditional Chinese medicine are fewer. Shennao Fuyuan Tang (SNFYT) is a Chinese medicine formula for the treatment of ischemic stroke, which was authorized by Professor Hu Guoheng, a famous Chinese medicine doctor in Hunan Province, China. The formula is based on Buyang Huanwu Decoction, which is composed of *Rehmanniae Radix Praeparata* (Shudihuang, SDH), *Cornus Officinalis Sieb*. *Et Zucc*. (Shanzhuyu, SZY), *Rhizoma Rhizoma Dioscoreae* (Shanyao, SY), *Hedysarum Multijugum Maxim*. (Hangqi, HQ), *Cortex Moutan* (Mudanpi, MDP), *Angelicae Sinensis Radix* (Danggui, DG), *Radix Paeoniae Rubra* (Chishao, CS), *Radix et Rhizoma Rhodiolae* (Hongjingtian, HJT), and *Pheretima* (Dilong, DL). The previous research group conducted a series of studies on the specific pharmacological mechanism of this formula, and the clinical trial results showed that SNFYT could significantly reduce the blood viscosity of patients and improve their clinical symptoms [10]. Animal experiments showed that this formula has anti-inflammatory [11], neurotrophic [11–13], antiapoptotic [14], cerebral protective [15], promoting neural and vascular regeneration and activating HPA axis, improving cerebral ischemia [15–18]. Cellular experiments showed that the drug-containing serum of this formula had a protective effect on the hypoxic and glucose-deficient PC12 cells, and this effect may be related to the inhibition of pyroptosis [19]. Although the research team has achieved certain results in the early stage, it is far from enough to explain the connotation of Traditional Chinese Medicine and the direct mechanism of modern pharmacology of SNFYT, and further research is needed.

**Figure 1:**
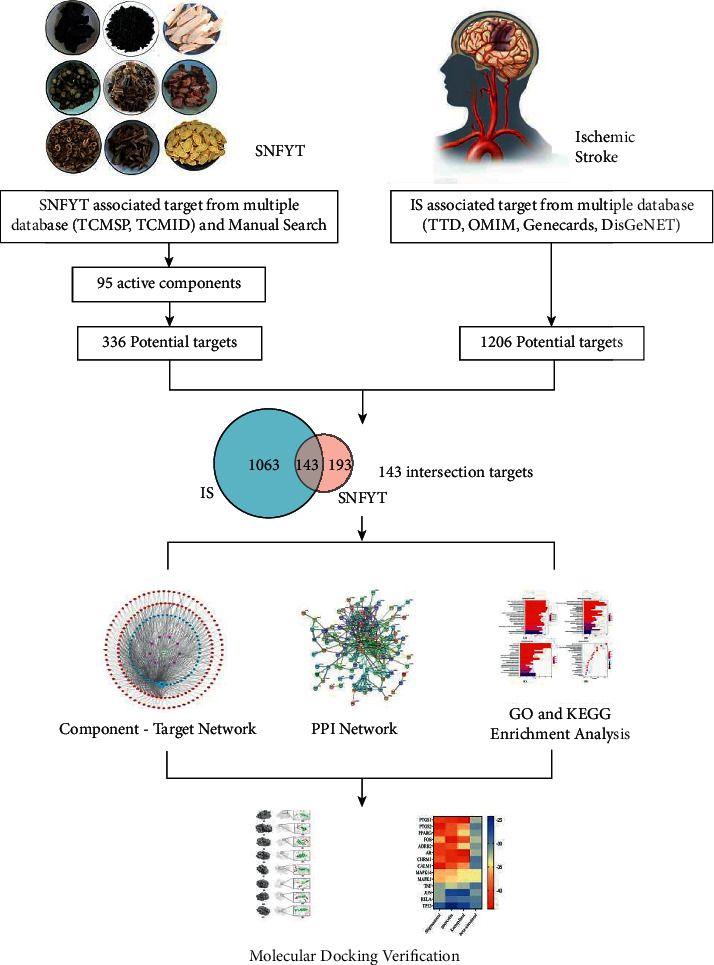
Flow chart of network pharmacology study on the mechanism of action of SNFYT in the treatment of IS.

Network pharmacology is based on the theory of systems biology and the fusion of computer technology to study the interaction of drugs, genes, proteins, etc., at the system level. The research concept of network pharmacology coincides with the holistic view of Traditional Chinese Medicine. It is used to predict and identify the biologically active components and targets of Chinese herbal medicine and to discover new indications through active components screening, target prediction, network construction, and analysis. The method has unique advantages and potential, which will help the public to further understand the role of Chinese medicine. This study aims to further reveal the potential mechanism of SNFYT in the treatment of ischemic stroke through network pharmacology and lays the foundation for future pharmacological and clinical research on ischemic stroke. The protocol of our experimental process is shown in Figure 1.

## 2. Methods

### 2.1. Establishment of a Database of SNFYT Target Genes and IS-Related Genes

The components of herbs of SNFYT were obtained from TCMSP (https://old.tcmsp-e.com/tcmsp.php) [20] and TCMID (https://119.3.41.228:8000/tcmid/) [21]. Screening criteria of the effective components were based on the oral bioavailability (OB) ≥30% and drug-likeness (DL) ≥0.18. Because *Pheretima* has too little active ingredient and related target information in the two databases mentioned above, the active ingredients and related targets were searched manually [22–24]. The UniProt database (https://www.uniprot.org/) [25] was adopted to change protein names to their corresponding gene symbols.

IS-related genes were collected from four databases: DrugBank database (https://go.drugbank.com/) [26], OMIM (https://www.omim.org/) [27], DisGeNET database (https://www.disgenet.org/) [28], and Therapeutic Target Database (TTD, https://db.idrblab.net/ttd/) [29]. We retrieved “Ischemic stroke, Cerebral infarction” as the keywords to obtain the targets of IS by four databases.

### 2.2. Integration of Candidate Targets of SNFYT for IS

The intersection between SNFYT-associated targets and IS-associated targets was considered as the candidate targets of SNFYT for treating IS. Hence, we obtained these candidate targets and generated a corresponding Venn diagram by using the R software (version 4.0.3).

### 2.3. Construction of Component-Target Network

Intersection genes of SNFYT-related targets and IS-related targets were entered into Cytospace software (version 3.7.2) as candidate targets to construct the component-target network. Degree and betweenness centrality are important topological parameters of networks and were calculated in Cytoscape to measure the topological importance of target and component nodes. The nodes were identified as core targets and core components according to the “degree” and “betweenness centrality” values, which were larger than the average degree and average betweenness centrality of all nodes in the network.

### 2.4. Construction of Protein-Protein Interaction (PPI) Network

To comprehensively screen the core targets, we use String 11.0 (https://string-db.org/) [30] to build protein-protein interaction (PPI) network. Totally, 143 core candidate targets were imported into the string database, the species was limited to “human,” the score value of protein interaction parameters was set to be greater than 0.9, the single node in the network was removed, other parameters remained unchanged, the results were saved in “tsv” file, the node and binding rate score information in the file were retained, and the PPI network was constructed by importing into Cytoscape (version 3.7.2).

### 2.5. Gene Ontology and Pathway Enrichment

The “cluster profiler” package, “colorspace” package, and “stringi” package of R software (version 4.0.3) were used to analyze the GO and KEGG enrichment annotations of candidate targets. The GO analysis included three levels of biological process, cell component, and molecular function, and the top 10 with the highest enrichment were selected, respectively. The top 20 signal pathways with the highest enrichment were also selected for KEGG analysis.

### 2.6. Molecular Docking Verification

Seven targets with the highest degree in the PPI network and seven core targets in the component-target core network were selected for molecular docking with four core components in the component-target core network. Download the “sdf” file of the 3D structure of the core component from the PubChem database (https://pubchem.ncbi.nlm.nih.gov/), imported it into the Chem3D software (version 19.0.0.22) for molecular structure energy optimization, and saved it as a mol2 format file from the PDB database (https://www.rcsb.org/) download the crystal structure of the core target protein, used Pymol software (version 4.6.0) to delete the water molecule of the target protein and the ligand located in the active pocket, and selected the small molecule ligand specific to the target protein as the active center. In the AutoDockTools software (version 1.5.6), added hydrogen atoms, calculate charges, set atoms, adjust charges and bond twists on small molecules, and saved the data in the “pdbqt” file. Used Autodock Vina [31] (version 1.1.2) to do small molecule and protein docking took the lowest scoring conformation and used Pymol (version 4.6.0) for analysis and mapping.

## 3. Results

### 3.1. Active Components of SNFYT

A total of 95 active components of 9 kinds of Chinese herbs with OB ≥30% and DL ≥0.18 were obtained from the databases and relevant literature, including 20 components from SZY, 16 components from SY, 20 components from HQ, 11 components from MDP, 29 components from CS, five components from HJT, eight components from DL, two components from SDH, and four components from DG (Table 1). In addition, sitosterol is a common component of SDH/SZY/MDP/CS, Stigmasterol is a common component of SDH/SZY/SY/DG/CS, ethyl oleate is a common component of SZY/CS, beta-sitosterol is a common component of SZY/DG/CS, Mairin is a common component of HQ/MDP, kaempferol and Quercetin are common components of HQ/MDP/HJT, paeoniflorin_qt, (+)-catechin, benzoyl, and paeoniflorin are common components of MDP/CS, and ellagic acid is a common component of CS/HJT. To make it easier to construct a visual network, we renumbered the components of the common target (Table 2).

### 3.2. Targets of SNFYT and IS

**Figure 2:**
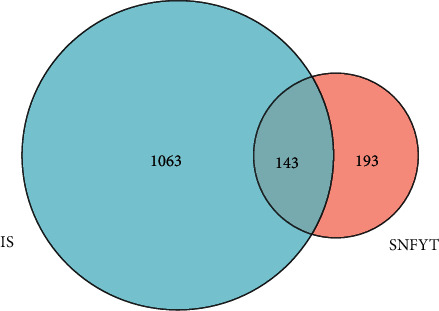
Intersection targets' venn diagram of SNFYT and IS.

A total of 336 targets were obtained through the TCMSP, TCMID, and manual retrieval. Using the four available resources, namely, TTD, DrugBank, Dis-GeNET, OMIM databases, we obtained 1206 IS-related targets. According to the candidate components and IS target genes, use R software to get their cross genes. Finally, 143 candidate genes were found, as shown in Figure 2.

### 3.3. Component-Target Network Analysis and Core Nodes Screening

**Figure 3:**
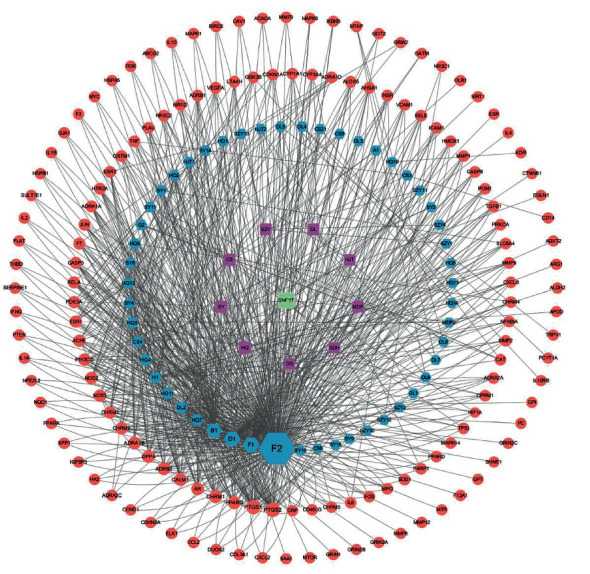
Component-target network. Green is the TCM formula, purple is an herb, blue is the component, and red is the gene target. The size of component nodes is proportional to the number of degrees.

Based on the above 95 active components, 143 SNFYT and IS cross-targets were collected from two databases, with no duplicate values. Use Cytoscape to establish a network of SNFYT-component-target, containing 204 nodes and 871 edges (Figure 3). To further determine the core nodes, use the Network analyzer function in Cytoscape 3.7.2 to analyze the nodes degree and betweenness centrality and obtain the median of genes, components, and herbs, respectively. The analysis results showed that the nine herbs median of DC = 10 and median of BC = 0.00824326, the 51 components median of DC = 23 and median of BC = 0.00391469, the 143 genes median of DC = 6 and median of BC = 0.000384.

**Figure 4:**
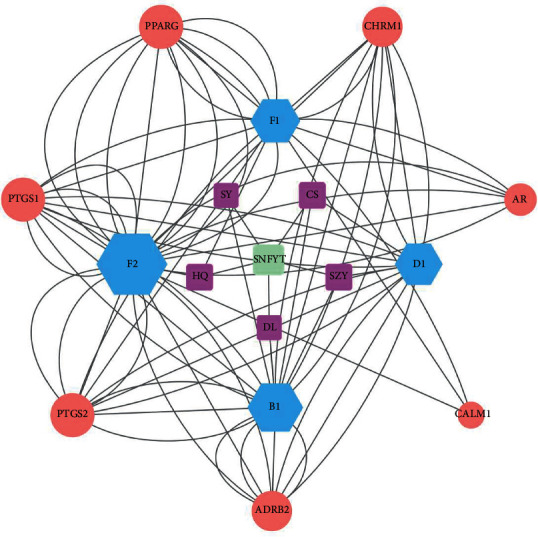
Component-target core network. Green is the TCM formula, purple is an herb, blue is the component, and red is the gene target. The size of component nodes is proportional to the number of degrees.

We screened herbs, components, and gene targets with DC >10 and BC >0.00824326, DC >46, and BC >0.00782938, DC > 36, BC > 0.00230646, respectively, to construct a component-gene core network. As shown in (Figure 4), component-target core network included 17 nodes (7 targets, four active components, five herbs) and 78 target-disease interactions. The first three components are F2, B1, D1, and the first three therapeutic targets are PTGS2, PTGS1, and PPARG. It was suggested that the mechanism of SNFYT for treating IS was closely related to these core components and targets. In summary, SNFYT played a cooperative role in treating IS through multiple potential targets.

### 3.4. PPI Network Analysis

**Figure 5:**
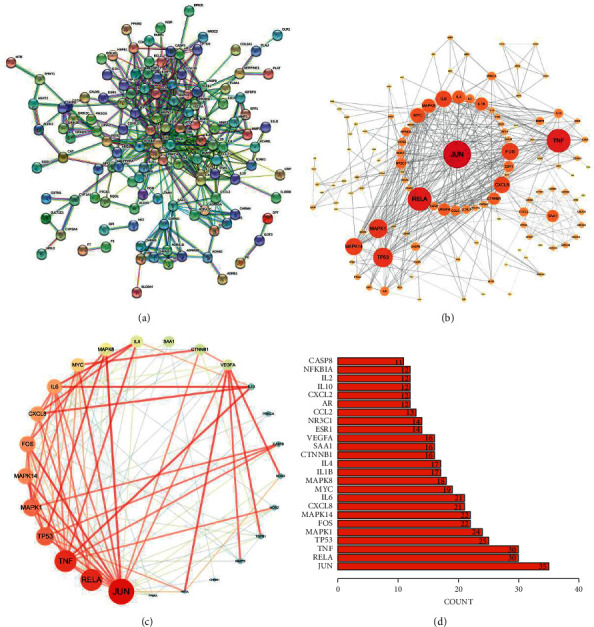
PPI network of candidate targets of SNFYT for IS: (a) construction of PPI network in SNFYT in treating IS using STRING database. (b) Use cytoscape to visualize the PPI network further. The node size and color represent the importance of the node in the network. (c) Topological analysis of potential targets in SNFYT in treating IS by using network analyzer. (d) The top 25 core genes visualization was obtained using R software according to the relevant number of nodes.

The 143 candidate targets were imported into the STRING database. The research species were limited to “Homo sapiens,” the “minimum required interaction score” was set to 0.9, and “hide disconnected nodes in the network.” The rest of the parameters remained the default values to obtain the protein-protein interaction network(PPI) between common targets (Figure 5(a)). Then, the new PPI network is obtained by analyzing the network with “generating styles from statistical tools” in Cytoscape software and setting the size and color of nodes to reflect the reactivity value and the thickness and color of lines to reflect the fusion score. The new PPI network (Figure 5(b)) is composed of 121 nodes and 468 edges. The average values of degree, betweenness centrality, and closeness centrality are 8, 0.018, and 0.338, respectively. Twenty-five targets meet the above-average values of the three, as shown in Table 3. The “tsv” file was used to run the “R” 4.0.2 software to draw a histogram (Figure 5(c)), and the first 25 targets of the number of nodes were selected to display as shown in Figure (Figure 5(d)). The top 10 targets with the highest degrees of freedom were Jun (degree = 35), TNF (degree = 30), RELA (degree = 30), TP53 (degree = 25), MAPK1 (degree = 24), FOS (degree = 22), MAPK14 (degree = 22), IL6 (degree = 21), CXCL8 (degree = 21), and MYC (degree = 19).

### 3.5. GO Enrichment Analysis

**Figure 6:**
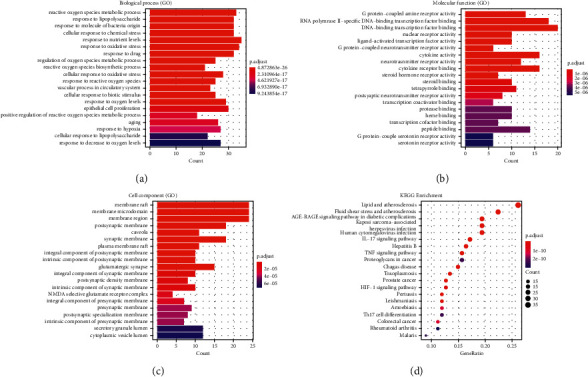
Enrichment analysis of candidate targets. (a) Barplot: biological process (GO enrichment analysis). (b) Barplot: molecular function (GO enrichment analysis). (c) Barplot: cell component (GO enrichment analysis). (d) Barplot: KEGG enrichment analysis.

Because the Clusterprofiler in R has high classification and enrichment accuracy and a visualization module for the results, it can be used to further study the target of SNFYT on IS. The above 143 targets are used for GO enrichment analysis and KEGG enrichment analysis. As shown in Figure 6, describes the first 20 GO terms of biological process (BP) (Figure 6(a)), molecular function (MF) (Figure 6(b)), and cell component (CC) (Figure 6(c)), respectively. Results covered that 143 targets of SNFYT for treatment on IS in BP were mainly associated with reactive oxygen species metabolic process and response to lipopolysaccharide. In the aspect of MF, mainly related to G protein-coupled amine receptor and genetic transcription, etc. In the aspect of CC, mainly related to the cell membrane.

### 3.6. KEGG Pathway Enrichment Analysis

**Figure 7:**
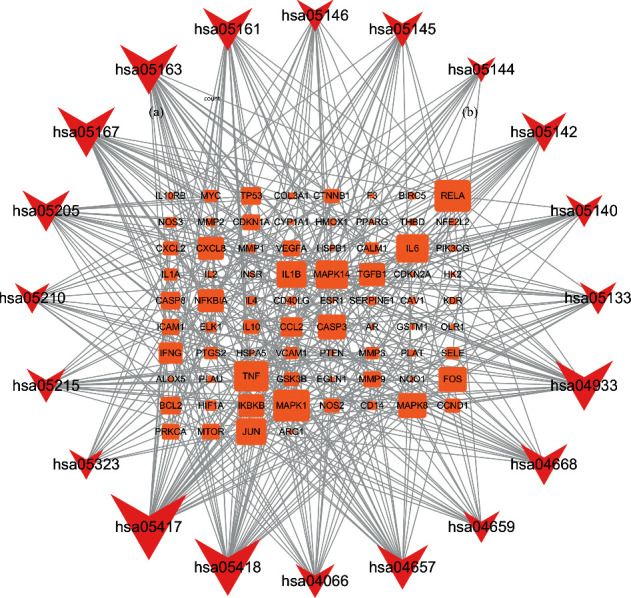
Target-pathway network. The orange nodes represent the target nodes. The brick-red nodes represent the corresponding pathways.

Using R software to perform KEGG enrichment analysis on 143 candidate genes, 170 results were obtained (*P* < 0.05). The top 20 pathways are shown in Figure 6(d), including the IL-17 signaling pathway, TNF signaling pathway, and HIF-1 signaling pathway. In addition, we have also constructed a target-pathway network to further prove the important role of core targets in signaling pathways (Figure 7). It can be seen that SNFYT plays a role in mediating anti-inflammatory, hypoxic perception, and response through multiple pathways and multiple targets, thereby treating IS on the overall level.

### 3.7. Molecular Docking Verification

**Figure 8:**
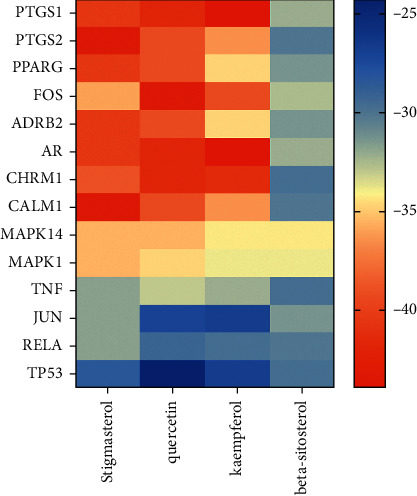
Molecular docking heat map of 4 core components and 14 core targets. The color indicates an affinity score. Red represents the lowest affinity score, the highest affinity between receptor and ligand, blue represents the highest affinity score and the lowest affinity between receptor and ligand.

**Figure 9:**
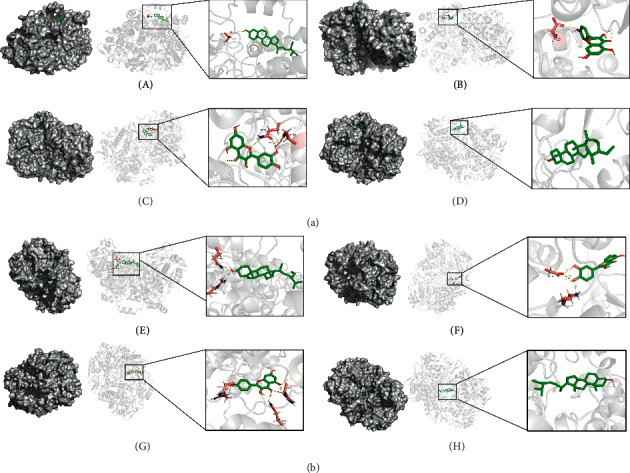
The docking mode of PTGS1 (a) and PTGS2 (b) with stigmasterol (A), quercetin (B), kaempferol (C), and beta-sitosterol (D), respectively.

The lower the binding energy, the more stable the binding of ligand and receptor. Therefore, the binding energy ≤−5.0 kJ/mol was used as the screening condition. In this study, the molecular docking results of four core components and 14 core targets are shown in Table 3 and Figure 8. The binding energy between them is far less than −5.0 kJ/mol, suggesting that the core components of SNFYT not only can bind to core targets but also has good binding power. The binding energy of PTSG1, PTSG2, PPARG, FOS, ADRB2, AR, CHRM1, and CALM1 with the stigmasterol, quercetin, and kaempferol is low. The molecular docking pattern is shown in Figure 9.

## 4. Discussion

Due to the level of technology in ancient China, Chinese medicine has developed a series of methods to understand the human body and treat diseases from a macroscopic level, which we call the holistic view. As a means to intervene in diseases under the guidance of the holistic view, Chinese medicine is a regulation of the overall state of the human body with multilevel, multipathway, and multitarget characteristics. In addition, stroke is also a very complex disease, mostly based on long-term cerebrovascular lesions. Sudden changes in blood pressure and blood flow lead to vascular obstruction and then cause a series of cascade reactions and changes in brain parenchyma. Therefore, both the multilevel, multipathway, and multitarget synergistic effects of herbal medicines and the complexity of stroke disease pathogenesis and evolution pose a great challenge to elucidate the mechanism of action of SNFYT for stroke disease from the molecular level [32]. Network pharmacology has updated the research paradigm from the previous “one target, one drug” model to a “network target, multiple components” model, which provides new ideas to elucidate the mechanism of action of SNFYT [33, 34]. Therefore, to further explore the active ingredients of SNFYT, predict the targets, and investigate the drug-gene-disease relationship, the mechanism of action of SNFYT was systematically described by network pharmacology technology and validated by molecular docking technique in this study.

We firstly identified 95 active components of SNFYT (Table 1) and then predicted 143 intersection targets of active components and stroke disease as the candidate targets of SNFYT for stroke disease (Figure 2). Next, we constructed a component-target network (Figure 3) based on the 143 candidate targets using Cytoscape and screened the four core components of SNFYT, which were F2 (quercetin), F1 (kaempferol), D1 (beta-sitosterol), and B1 (stigmasterol) (Figure 4). Relevant basic studies also confirm the role of the above components, and Quercetin is a common component of *Hedysarum Multijugum Maxim*., *Cortex Moutan*, *Radix et Rhizoma Rhodiolae*, widely found in plants such as fruits, vegetables, and cereals, and is one of the most important dietary antioxidants [35]. Basic studies have shown that quercetin also has anti-inflammatory [36], antiapoptotic [37], antioxidative stress damage [38], promotes angiogenesis [38], and prevents atherosclerosis [39, 40], with targets such as iNOS, caspase-3, antioxidant enzymes, Nrf2, MMP-9, NF-*κ*B, and PARP [39, 41–46], involving signaling pathways such as PGC-1 alpha [47], NF-*κ*B [48], and Akt. Kaempferol is also a common component of *Hedysarum Multijugum Maxim*., *Cortex Moutan*, *Radix et Rhizoma Rhodiolae* It has anti-inflammatory [49], antioxidant [50], and apoptosis inhibitory [51] effects and is closely related to the NF-*κ*B signal pathway [52]. Beta-Sitosterol is a common component of *Cornus Officinalis Sieb*. *Et Zucc*, *Angelicae Sinensis Radix*, *Radix Paeoniae Rubra*, and promotes neointima formation in the brain of gerbils with ischemia/reperfusion injury promoting the expression of von Willebrand factor, vascular endothelial growth factor (VEGF), VEGF receptor Flk-1 and vascular stromal adhesion protein [53]. Stigmasterol is *Rehmanniae Radix Praeparata*, *Cornus Officinalis Sieb*. *Et Zucc*., *Rhizoma Rhizoma Dioscoreae*, *Angelicae Sinensis Radix*, Radix a common component of Paeoniae Rubra, can achieve neuroprotective effects by regulating cellular autophagy [54, 55], a process that may be related to AMPK/mTOR and JNK signaling pathways. It also inhibits the expression of GluN2B and attenuates excitotoxicity and oxidative stress [54].

Based on the degree value from largest to smallest, seven core targets were selected from the component-target core network (Figure 4), which were PTGS2, PTGS1, PPARG, CHRM1, ADRB2, CALM1, and AR. In addition, seven core targets were selected based on the PPI protein interaction network, in descending order of magnitude: Jun, TNF, RELA, TP53, MAPK1, FOS, and MAPK14. Therefore, a total of 14 core targets were selected.

PTGS is a key enzyme in the synthesis of prostaglandins and affects platelet aggregation. PTGS1 and PTGS2 are two isozymes of PTGS, which differ greatly in expression regulation and tissue distribution. PTGS2 acts only when stimulated by cytokines, growth factors, NO, etc., and is mainly involved in the inflammatory response, also known as the inducible enzyme [56]. It has been shown that PTGS2 is associated with carotid plaque, platelet activation, and TXA2 levels [57] and is a target for the effects of smoking and alcohol consumption on stroke [58], and inhibition of PTGS2 can have a protective effect on MCAO mice through the NF-*κ*B signaling pathway [59]. PPARG plays an important role in the inflammatory response after ischemic stroke and can target EGR-1 to inhibit the inflammatory response after ischemic stroke [60], and its activation inhibition can increase M2 and decrease M1 microglia/macrophage [61, 62]. In addition, PPARG plays a role in angiogenesis after ischemic stroke and can promote functional recovery [63]. ADRB2 is an important regulator of the neuroimmune response after ischemic stroke and affects the inflammatory response of microglia/macrophages [64], and inhibition of ADRB2-mediated upregulation of HIF-1*α* can reduce blood-brain barrier damage during acute cerebral ischemia [65]. CALM1 mediates the regulation of multiple proteins through calcium binding, and correlation analysis has shown that CALM1 is associated with blood clotting in patients with ischemic stroke [66]. AR is a ligand-activated transcription factor that mainly affects cell proliferation and differentiation in target tissues, and a study has demonstrated that overexpression of AR is neuroprotective in MCAO mice [67], potentially contributing to gender differences in human stroke outcomes [68]. JUN, a member of the AP-1 family of transcription factors, also known as c-jun, is a downstream target of Jnk [69] and is associated with apoptosis [70], inflammatory response [71], and reperfusion injury during stroke disease [72]. TNF, also known as TNF-alpha, is secreted mainly by macrophages and is capable of mediating certain tumor cell death, is an important inflammatory mediator after ischemic stroke, can affect the permeability of the blood-brain barrier, and is closely associated with neurotoxicity [73, 74]. The protein P65 expressed by RELA is a key molecule in the NF-*κ*B signaling pathway and can inhibit microglia M1 polarization and promote M2 polarization, thus achieving neuroprotective effects [75, 76]. TP53 is associated with the cell cycle, and TP53 is related to the cell cycle and is an oncogene. Related studies have confirmed that TP53 is closely related to the pathological process of ischemic stroke [77] and is also associated with functional prognosis after stroke [78]. MAPK1, also called ERK2, is an important molecule in the MAPK signaling pathway, and ERK2 is associated with platelet aggregation [79], and inhibition of ERK2 can inhibit apoptosis after ischemic stroke [80]. Some studies have reported that MAPK14 can be used as a marker of cardiogenic stroke [81], and it has also been demonstrated from large data of ischemic stroke-related LncRNA that MAPK14 is a key gene in ischemic stroke, but there are fewer relevant animal experimental studies. Studies on CHRM1 and FOS related to ischemic stroke are very rare, but this just provides new ideas for us to study ischemic stroke. In summary, most of the core targets are related to the inflammatory response after ischemic stroke, suggesting that SNFYT may exert anti-inflammatory effects through multiple pathways.

GO enrichment analysis showed that the biological processes involved in SNFYT are mainly focused on the body's response to oxidative stress and nutrient levels, and the cellular components involved are mainly cell membrane and endosomal systems, and the molecular functions are mainly DNA transcription. KEGG enrichment analysis showed that SNFYT prevention and control of IS mainly involves IL-17 signaling pathway, TNF signaling pathway, HIF-1 signaling pathway, IL-17 signaling pathway, and TNF signaling pathway, which are all important inflammatory pathways, and studies have shown that 70%–80% of nerve damage is associated with inflammatory responses after stroke [82]. Both IL-17 and TNF are thought to play an important role in the inflammatory cascade response in ischemic stroke [83–86], so both the IL-17signaling pathway and TNF signaling pathway are intimately involved in the pathological evolution of IS. Cellular perception and response to oxygen is another very important process in the pathological evolution of ischemic stroke, and the HIF-1 signaling pathway plays an important role in this process. Under normoxic conditions, HIF-1*α* is degraded by E3 ubiquitin ligases containing pVHL [87], and under hypoxic conditions, the HIF1-*α* subunit is not recognized by pVHL and can accumulate in large amounts to bind to *β* to form HIF-1 [88], which is widely expressed in mammals and is capable of mediating the transcription of more than 200 genes [89], affecting angiogenesis, cell differentiation, metabolism, and apoptosis [90]. Interestingly, the HIF-1 signaling pathway also plays an important role in inflammation [91] and interacts with the IL-17 signaling pathway and the TNF signaling pathway. It has been shown that enhanced STAT3 phosphorylation under hypoxic conditions promotes not only TH17 development [92] but also enhances HIF-1 transcription [93], which in turn can activate ROR*γ*t, thereby further accelerating TH17 maturation and differentiation [94]. Furthermore, it has been demonstrated that blocking the accumulation of HIF-1*α* can reduce the expression of TNF-*α* and IL-6 and increase the expression of IL-10 [95, 96], that the expression of TNF-*α* in CD8^+^ T cells lacking HIF-1a is subsequently reduced [96], and that HIF-1*α* can indirectly regulate TNF-*α* production by macrophages [97]. These results further suggest that SNFYT may exert therapeutic effects through the IL-17 signaling pathway, TNF signaling pathway, and HIF-1 signaling pathway regulating the inflammatory cascade response after ischemic stroke.

## 5. Conclusion

In summary, we have explored the core components and core targets of SNFYT using network pharmacology and molecular docking methods and elucidated that the intervention of SNFYT on the inflammatory cascade of IS may be a potential pathway of action of SNFYT in the treatment of IS, laying an experimental foundation for further study of the “multicomponent, multitarget, and multichannel” action of SNFYT in the treatment of IS.

## Figures and Tables

**Table 1:** Active components of SNFYT.

Herb	Mol ID	Molecule name	MW	OB (%)	DL
SZY	MOL001494	Mandenol	308.56	42.00	0.19
SZY	MOL001495	Ethyl linolenate	306.54	46.10	0.20
SZY	MOL001771	Poriferast-5-en-3beta-ol	414.79	36.91	0.75
SZY	MOL002879	Diop	390.62	43.59	0.39
SZY	MOL003137	Leucanthoside	462.44	32.12	0.78
SZY	MOL005360	Malkangunin	432.56	57.71	0.63
SZY	MOL005481	2,6,10,14,18-Pentamethylicosa-2,6,10,14,18-Pentaene	342.67	33.40	0.24
SZY	MOL005486	3,4-Dehydrolycopen-16-al	548.92	46.64	0.49
SZY	MOL005489	3,6-Digalloylglucose	484.40	31.42	0.66
SZY	MOL005503	Cornudentanone	378.56	39.66	0.33
SZY	MOL005530	Hydroxygenkwanin	300.28	36.47	0.27
SZY	MOL005531	Telocinobufagin	402.58	69.99	0.79
SZY	MOL008457	Tetrahydroalstonine	352.47	32.42	0.81
SZY	MOL000554	Gallic acid-3-O-(6′-O-galloyl)-glucoside	484.40	30.25	0.67
SZY	MOL005552	Gemin D	634.49	68.83	0.56
SZY	MOL005557	Lanosta-8, 24-dien-3-ol, 3-acetate	468.84	44.30	0.82
SY	MOL001559	Piperlonguminine	273.36	30.71	0.18
SY	MOL001736	(-)-Taxifolin	304.27	60.51	0.27
SY	MOL000310	Denudatin B	356.45	61.47	0.38
SY	MOL000322	Kadsurenone	356.45	54.72	0.38
SY	MOL005429	Hancinol	372.50	64.01	0.37
SY	MOL005430	Hancinone C	400.51	59.05	0.39
SY	MOL005435	24-Methylcholest-5-enyl-3belta-O-glucopyranoside_qt	400.76	37.58	0.72
SY	MOL005438	Campesterol	400.76	37.58	0.71
SY	MOL005440	Isofucosterol	412.77	43.78	0.76
SY	MOL005458	Dioscoreside C_qt	444.72	36.38	0.87
SY	MOL000546	Diosgenin	414.69	80.88	0.81
SY	MOL005461	Doradexanthin	584.96	38.16	0.54
SY	MOL005463	Methylcimicifugoside_qt	556.81	31.69	0.24
SY	MOL005465	AIDS180907	394.45	45.33	0.77
SY	MOL000953	CLR	386.73	37.87	0.68
HQ	MOL000239	Jaranol	314.31	50.83	0.29
HQ	MOL000296	Hederagenin	414.79	36.91	0.75
HQ	MOL000033	(3S, 8S, 9S, 10R, 13R, 14S, 17R)-10,13-Dimethyl-17-[(2R, 5S)-5-propan-2-yloctan-2-yl]-2, 3, 4, 7, 8, 9, 11, 12, 14, 15, 16, 17-dodecahydro-1H-cyclopenta [a] phenanthren-3-ol	428.82	36.23	0.78
HQ	MOL000354	Isorhamnetin	316.28	49.60	0.31
HQ	MOL000371	3, 9-di-O-methylnissolin	314.36	53.74	0.48
HQ	MOL000374	5′-Hydroxyiso-muronulatol-2′, 5′-di-O-glucoside	642.67	41.72	0.69
HQ	MOL000378	7-O-methylisomucronulatol	316.38	74.69	0.30
HQ	MOL000379	9, 10-Dimethoxypterocarpan-3-O-*β*-D-glucoside	462.49	36.74	0.92
HQ	MOL000380	(6aR, 11aR)-9, 10-Dimethoxy-6a, 11a-dihydro-6H-benzofurano [3, 2-c] chromen-3-ol	300.33	64.26	0.42
HQ	MOL000387	Bifendate	418.38	31.10	0.67
HQ	MOL000392	Formononetin	268.28	69.67	0.21
HQ	MOL000398	Isoflavanone	316.33	109.99	0.30
HQ	MOL000417	Calycosin	284.28	47.75	0.24
HQ	MOL000433	FA	441.45	68.96	0.71
HQ	MOL000438	(3R)-3-(2-hydroxy-3, 4-dimethoxyphenyl) chroman-7-ol	302.35	67.67	0.26
HQ	MOL000439	Isomucronulatol-7, 2′-di-O-glucosiole	626.67	49.28	0.62
HQ	MOL000442	1, 7-Dihydroxy-3, 9-dimethoxy pterocarpene	314.31	39.05	0.48
MDP	MOL007369	4-O-methylpaeoniflorin_qt	332.38	67.24	0.43
MDP	MOL007374	5-[[5-(4-Methoxyphenyl)-2-furyl] methylene] barbituric acid	312.30	43.44	0.30
MDP	MOL007382	Mudanpioside-h_qt 2	336.37	42.36	0.37
MDP	MOL007384	Paeonidanin_qt	330.41	65.31	0.35
CS	MOL001918	Paeoniflorgenone	318.35	87.59	0.37
CS	MOL001921	Lactiflorin	462.49	49.12	0.80
CS	MOL001924	Paeoniflorin	480.51	53.87	0.79
CS	MOL002714	Baicalein	270.25	33.52	0.21
CS	MOL002776	Baicalin	446.39	40.12	0.75
CS	MOL004355	Spinasterol	412.77	42.98	0.76
CS	MOL006990	(1S, 2S, 4R)-trans-2-Hydroxy-1, 8-cineole-B-D-glucopyranoside	332.44	30.25	0.27
CS	MOL006992	(2R, 3R)-4-methoxyl-distylin	318.30	59.98	0.30
CS	MOL006994	1-O-beta-d-glucopyranosyl-8-o-benzoylpaeonisuffrone_qt	302.35	36.01	0.30
CS	MOL006996	1-O-beta-d-glucopyranosylpaeonisuffrone_qt	332.38	65.08	0.35
CS	MOL006999	Stigmast-7-en-3-ol	414.79	37.42	0.75
CS	MOL007004	Albiflorin	480.51	30.25	0.77
CS	MOL007005	Albiflorin_qt	318.35	48.70	0.33
CS	MOL007008	4-Ethyl-paeoniflorin_qt	332.38	56.87	0.44
CS	MOL007012	4-O-methyl-paeoniflorin_qt	332.38	56.70	0.43
CS	MOL007014	8-Debenzoylpaeonidanin	390.43	31.74	0.45
CS	MOL007016	Paeoniflorigenone	318.35	65.33	0.37
CS	MOL007018	9-Ethyl-neo-paeoniaflorin A_qt	334.40	64.42	0.30
CS	MOL007022	EvofolinB	318.35	64.74	0.22
CS	MOL007025	Isobenzoylpaeoniflorin	584.62	31.14	0.54
CS	MOL005043	Campest-5-en-3beta-ol	400.76	37.58	0.71
HJT	MOL002823	Herbacetin	302.25	36.07	0.27
HJT	MOL004020	Gossypetin	318.25	35.00	0.31
DL	—	Leucinum	—	—	—
DL	—	GLY	—	—	—
DL	—	L-valin	—	—	—
DL	—	Palmitic acid	—	—	—
DL	—	EIC	—	—	—
DL	—	Adenine	—	—	—
DL	—	HX	—	—	—
DL	—	GUN	—	—	—
SDH/CS/MDP/SZY	MOL000359	Sitosterol	414.79	36.91	0.75
SDH/CS/DG/					
SY/SZY	MOL000449	Stigmasterol	412.77	43.83	0.76
SZY/CS	MOL002883	Ethyl oleate (NF)	310.58	32.40	0.19
SZY/DG/CS	MOL000358	Beta-sitosterol	414.79	36.91	0.75
HQ/MDP	MOL000211	Mairin	456.78	55.38	0.78
HQ/MDP/HJT	MOL000422	Kaempferol	286.25	41.88	0.24
HQ/MDP/HJT	MOL000098	Quercetin	302.25	46.43	0.28
MDP/CS	MOL001925	paeoniflorin_qt	318.35	68.18	0.40
MDP/CS	MOL000492	(+)-Catechin	290.29	54.83	0.24
MDP/CS	MOL007003	Benzoyl paeoniflorin	584.62	31.14	0.54
CS/HJT	MOL001002	Ellagic acid	302.20	43.06	0.43

**Table 2:** List of common active Components in SNFYT.

Herb	Number	Mol ID	Molecule name
SDH/SZY/MDP/CS	A1	MOL000359	Sitosterol
SDH/SZY/SY/DG/CS	B1	MOL002045	Stigmasterol
SZY/CS	C1	MOL002883	Ethyl oleate (NF)
SZY/DG/CS	D1	MOL000358	Beta-sitosterol
HQ/MDP	E1	MOL000211	Mairin
HQ/MDP/HJT	F1	MOL000422	Kaempferol
HQ/MDP/HJT	F2	MOL000098	Quercetin
MDP/CS	G1	MOL001925	Paeoniflorin_qt
MDP/CS	G2	MOL000492	(+)-Catechin
MDP/CS	G3	MOL007003	Benzoyl paeoniflorin
CS/HJT	H1	MOL001002	Ellagic acid

**Table 3:** The result of core components docked with components.

Component	Molecular formula	CAS	Binding energy (kJ/mol)													
			PTGS1	PTGS2	PPARG	CHRM1	AR	CALM1	ADRB2	JUN	RELA	TNF	TP53	MAPK1	MAPK14	FOS
Stigmasterol	C_29_H_48_O	83-48-7	−40.58	−43.51	−40.59	−38.91	−40.58	−43.51	−40.58	−31.80	−31.80	−31.80	−28.45	−35.56	−35.56	−35.98
Quercetin	C_15_H_10_O_7_	117-39-5	−41.84	−39.33	−39.32	−41.84	−41.84	−39.33	−39.33	−27.20	−29.29	−33.05	−24.27	−34.73	−35.56	−43.51
Kaempferol	C_15_H_10_O_6_	520-18-3	−43.93	−36.40	−34.72	−41.42	−43.93	−36.40	−34.73	−26.78	−29.71	−32.22	−26.78	−33.89	−34.31	−39.33
Beta-sitosterol	C_29_H_50_O	83-46-5	−32.22	−30.12	−31.38	−29.71	−32.22	−30.12	−31.38	−31.38	−30.12	−29.71	−29.71	−33.89	−34.31	−32.64

## Data Availability

All the data used to support the findings of this study are available in the article.
